# A three-dimensional (3D) printed simulator as a feasible assessment tool for evaluating hip arthroscopy skills

**DOI:** 10.1007/s00167-022-07125-w

**Published:** 2022-08-29

**Authors:** Bohong Cai, Shengfeng Duan, Jiahui Yi, Boon Huat Bay, Jiaxing Huang, Wei Huang, Ning Hu, Cheng Chen

**Affiliations:** 1 Department of Industrial and Product Design, School of Design, Sichuan Fine Arts Institute, Chongqing, China; 2 Department of Anatomy, Yong Loo Lin School of Medicine, National University of Singapore, Singapore, Singapore; 3 Department of Orthopedics, The First Affiliated Hospital of Chongqing Medical University, Chongqing, China

**Keywords:** Hip arthroscopy, Assessment, Medical simulation, 3D printing

## Abstract

**Purpose:**

The aims of this study were (1) to develop a three-dimensional (3D) printed simulator that facilitates the simulation of surgical skills for portal placement, intra-articular identification of anatomical structures and arthroscope navigation for hip arthroscopy and (2) to concurrently examine the feasibility of using this simulator as an assessment tool to evaluate trainees’ surgical competencies.

**Methods:**

A simulator was developed using a combination of medical imaging, computer-aided design, and 3D printing. A cross-sectional study was conducted with 29 participants divided into 3 subgroups (novice, intermediate and experienced). All participants performed related skills on the simulator, and their performance was evaluated using different assessment parameters. The participants’ qualitative feedback regarding the simulator was also collected. The data collated from each group of participants were subsequently compared.

**Results:**

Significant differences were observed between the three subgroups of participants with regard to the total checklist score (*F*_2,26_ = 11.3), total Arthroscopic Surgical Skill Evaluation score (*F*_2,26_ = 92.1), overall final global rating scale score (*F*_2,26_ = 49), number of times the participants used fluoroscopy (*F*_2,26_ = 7.4), and task completion times (*F*_2,26_ = 23.5). The participants’ performance in the simulated operation was correlated with their prior clinical experience. There was mainly positive feedback with regard to the fidelity and utility of the simulator in relation to the surgeons’ prior clinical experience.

**Conclusions:**

This study demonstrated that a reliable hip arthroscopic simulator can be developed for use by orthopedic surgeons to evaluate their hip arthroscopic skills before performing actual surgical operations.

**Level of evidence:**

Level III.

**Supplementary Information:**

The online version contains supplementary material available at 10.1007/s00167-022-07125-w.

## Introduction

Arthroscopy has been used as a routine operative method to treat many intra-articular conditions of the hip, such as femoroacetabular impingement and labral tears [5, 8, 21]. However, some of the critical skills of hip arthroscopy pose challenges to surgeons performing this technique, such challenges including portal placement, identification of anatomical structures and navigation of arthroscopy in the intra-articular space [11, 14, 27]. Lack of competence in these skills may translate into prolonged surgical time and bring about iatrogenic injuries [9, 10, 14, 29], which will seriously affect the overall surgical quality. Conventionally, surgeons need to spend years in the operating room to achieve the optimal level of arthroscopic skills.

Currently, the gap between the level of surgical skills required for a positive clinical outcome and that attained by less-skilled surgeons can be bridged to some extent by medical simulation [13, 16], which allows surgical trainees to practice and evaluate their clinical skills in an objective and systematic manner. As a widely recognized technology in the medical field, 3D printing has enormous potential in enhancing the development of task-specific simulators [7, 17, 20]. By combining relevant techniques, 3D printed models with precise anatomical structures, which are able to provide tactile sensation, can be created to facilitate simulation-based training and assessment [18, 19, 22], which would be beneficial for hip arthroscopic training.

Although various technologies have been applied to develop simulators that can facilitate the training of arthroscopic skills and allow trainees to demonstrate their proficiency in a risk-free environment [2, 3, 6, 23, 25], there is currently a lack of effective simulators that can replicate some of the important steps for hip arthroscopy, such as portal placement. In this context, providing a suitable and effective method for a novice arthroscopic surgeon to learn and assess important and requisite skills will improve clinical outcomes. Hence, the purposes of the present study were to develop a 3D printed simulator to simulate hip arthroscopic surgery, including portal placement, intra-articular identification of anatomical structures and arthroscope navigation, with concurrent validation of the simulator as an assessment tool. It has been hypothesized that by combining the techniques of medical imaging, computer-aided design (CAD) and 3D printing, an effective physical simulator can be developed to simulate critical steps involved in hip arthroscopy. Additionally, the performance of arthroscopic surgeons during the simulation could also possibly be correlated to their performance in the surgical operating room.

## Materials and methods

### Ethics approval

This study was reviewed and approved by the Research Ethics Committee of Chongqing Medical University, reference 2021-048. Informed consent was obtained from each participant. The validation study was conducted at a single academic training center.

### Development of the simulator

As computed tomography (CT) scans are able to show bony structures precisely [28], this modality was selected as the imaging resource for reconstructing the 3D structure of the hip joint. The deidentified CT data of the right hip joint from a 31-year-old female volunteer were used. The alpha angle and lateral center edge angle for the volunteer’s hip joint were 60.9° and 35.4°, respectively. The CT scanning (BrightSpeed, 16-slice CT scanner; General Electric, Boston, MA) was conducted in the horizontal plane with a slice width of 0.35 mm. The CT scans were saved as DICOM format data using InVesalius software, version 3.1.1 (Renato Archer Information Technology Center, Campinas, Brazil), to reconstruct 3D volumetric data, which were imported into a CAD file with the STereoLithography format. Next, the CAD file was imported into Meshmixer software, version 3.5 (Autodesk, San Rafael, CA) for 3D segmentation, which removes unwanted structures in the image. In the process of segmentation, only a minimum smoothing algorithm was applied to retain the anatomical features of the scanned region.

**Fig. 1:**
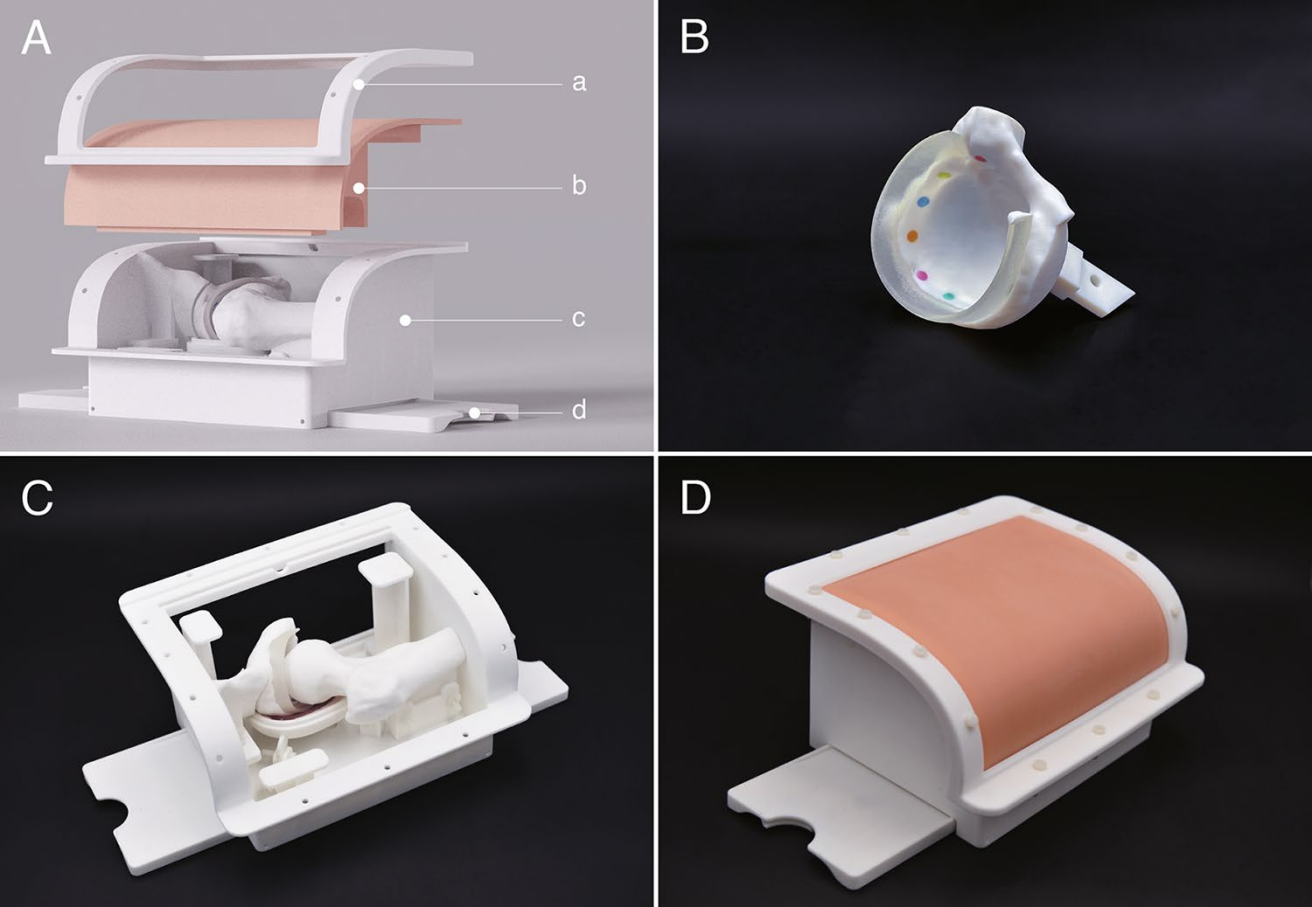
Design of the simulator. **A** Main components of the simulator: (a). clip frame to fix the soft component, (b). soft component to simulate the soft tissue, (c). container for holding all the bony and supporting structures, (d). bench fixer used in tandem with a bench vise to secure the simulator onto the table (foldable); **B** The 3D printed component of the acetabulum; **C** The 3D printed bony structures of the simulator; **D** Assembled simulator with the silicon component

For the design process, the CAD file was imported into Rhino software, version 6 (Rhinoceros, Seattle, WA). During this stage, unnecessary bony structures were removed, retaining only the anterior superior iliac spine, the acetabulum, and the proximal end of the femur. Since the volunteer was CT scanned in a normal lying position, the femoral structure was repositioned to simulate the hip in the tractional condition to enable access to the central compartment for the simulated operation [23, 28]. The box-shaped simulator was designed on the basis of a modular concept and comprised two main parts, one part as the soft component to simulate soft tissues and the other part as the hard component to simulate bony structures (Fig. 1A).

The structural framework of the anterior superior iliac spine, the acetabulum, and the proximal end of the femur were designed as independent modules, which allowed them to be replaced when simulating various hip conditions. The hip joint capsule was designed with a water drop shape, gradually enlarging in form from the femoral head to the acetabulum. This would coordinate with the traction force applied to the hip joint. As the acetabular labrum was not clearly defined in the CT scan, it was designed manually to have a 3–4 mm thickness with a width of approximately 8 mm. Nine fixed markings were incorporated on the surface of the acetabulum from the 8 o’clock position to the 4 o’clock position to aid the intra-articular identification of anatomical structures (Fig. 1B).

The main body of the simulator, including the anterior superior iliac spine, the proximal end of the femur, and the container with all the structural components, was 3D printed using an EOS Formiga P100 3D printer (EOS GmbH Electro Optical Systems, Krailling, Germany) and fabricated with white polyamide EOS PA2200 (Fig. 1C). The components of the acetabulum and the acetabular labrum were 3D printed as a single piece using a Stratasys J750 3D printer (Stratasys, Minneapolis, MN) and fabricated using VeroPureWhite, VeroCyanV, VeroMagentaV, VeroYell and Agilus materials. The soft component was fabricated in a silicon material, Ecoflex 00-30 (Smooth-on, Macungie, PA), using a mold that was designed and 3D printed (Fig. 1D).

### Hip arthroscopic simulator

**Fig. 2:**
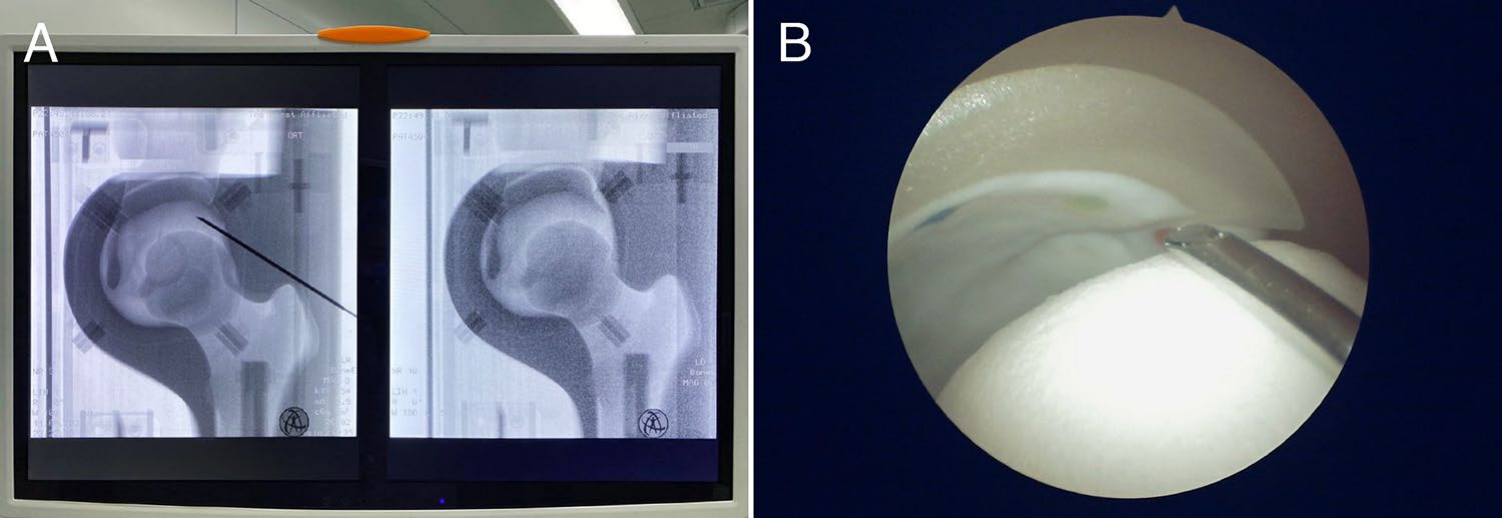
Usage of the 3D printed simulator. **A** Fluoroscopic images for the portal placement process; **B** Arthroscopic view of the process for establishing the distal anterolateral (DALA) portal

The simulator that was developed could be secured onto the table by using two bench vises. The materials of the simulator were photosensitive to radiography, and all the internal structures were visible by fluoroscopy (Fig. 2A). The anatomical landmarks of the anterior superior iliac spine and the greater trochanter could be identified by feeling the simulated soft tissue. When viewed using the arthroscopic camera, the intra-articular anatomical structures of the simulator would appear similar to those observed in the hip joint of the human body (Fig. 2B).

### Design of the validation study

To evaluate the validity of the simulator as an assessment tool, a cross-sectional study was conducted. Twenty-nine orthopedic surgeons from 11 hospitals were invited to participate in the validation study. All the participants were male and right-hand dominant, with ages ranging from 23 to 56 years old. They provided information regarding their previous experience in the different types of arthroscopic surgeries. In general, participants were divided into three subgroups based on their training level and experience in arthroscopy, viz*.*, junior doctors pursuing a higher degree and residents undergoing specialty training were classified as the novice group, junior specialist surgeons were classified as the intermediate group, and consultant surgeons and senior consultant surgeons were classified as the experienced group; their relevant experience in arthroscopy of the different joints is shown in Table 1. However, two junior specialist surgeons who had significantly less arthroscopy experience than their peers were categorized into the novice group. One consultant surgeon who had no prior experience in hip arthroscopy was placed into the intermediate group. Based on power calculation for one-way ANOVA comparing three groups, a sample size of six was needed in each group to obtain a power of 0.8 when the effect size was 0.9 and a significance level of 0.05 was employed.

**Table 1:** Overall Clinical experience of the subgroups

	Novice group (mean, 95% CI)	Intermediate group (mean, 95% CI)	Experienced group (mean, 95% CI)
*n*	12	9	8
Assisted hip arthroscopic surgery	4.3 [1.2, 7.3]	12 [1.6, 22.4]	67.5 [44.1, 90.9]
Performed hip arthroscopic surgery	0	0	30.6 [11,5.3]
Assisted arthroscopic shoulder labral repair	54.2 [7.7, 101]	106 [67.4, 144]	159 [57.9, 260]
Performed arthroscopic shoulder labral repair	11 [3.7, 18.3]	60 [21.8, 98.2]	130 [22.1, 238]
Assisted knee arthroscopic surgery	100 [42.7, 157]	199 [94.2, 304]	375 [174, 576]
Performed knee arthroscopic surgery	26.3 [12.1, 40.6]	149 [61.8, 236]	181 [79.9, 283]

Before the start of the simulated operation, a 4-h didactic lecture on hip arthroscopic surgery was provided to the participants. The contents covered in the lecture included relevant anatomical and operational knowledge, which was intended to help participants familiarize themselves with the steps that they would be performing on the simulator. For the simulated operation, the demo version of an arthroscope was used, with other standard surgical equipment and tools, such as fluoroscopic equipment, to enhance the accuracy of the simulation.

An arthroscopic surgeon with 2 years of experience performing hip arthroscopy assumed the role of an instructor to guide participants in performing tasks with the simulator. A task-specific checklist (Supplemental Table 1), created by two experienced staff surgeons based on an earlier study [25], was used to guide and evaluate the operational processes. The instructor graded the performance of participants using this task-specific checklist. The time taken for task completion and the number of times fluoroscopy was used were also noted by the instructor. Hand and arthroscopic footage for each simulated operation were videotaped. Another arthroscopic surgeon with 5 years of experience in hip arthroscopy served as a blinded assessor to review the deidentified videos after the study and graded each operation using the Arthroscopic Surgical Skill Evaluation Tool (ASSET) [15] and final global rating scale (GRS) [12]. After the simulated operation, a poststudy qualitative survey using a five-point Likert scale with anchor statements was conducted to collect feedback from the participants about their perception and utility of the simulator (Supplemental Table 2).

### Statistical analysis

The total scores for the task-specific checklist, ASSET, final GRS and feedback (with maximum scores of 15, 38, 5 and 20, respectively) were collated, and mean scores were calculated. The internal consistency and reliability for the task-specific checklist and ASSET scores were measured using Cronbach’s alpha. The differences in performance and feedback scores between the three groups of participants were assessed using one-way ANOVA with a post hoc Tukey test. The Pearson correlation coefficient was used to calculate the correlation between the task-specific checklist, ASSET and final GRS scores. The effect size was measured by Cohen's *f* statistic for one-way ANOVA. All statistical analyses were performed using R software, version 4.1.1 (R Foundation).

## Results

All participants performed the required operational steps on the 3D printed simulator. Cronbach’s alpha values of 1 for the total ASSET score and 0.7 for the task-specific checklist indicate good internal consistency/reliability. Cohen’s *f* values for the task-specific checklist, ASSET and final GRS were 0.9, 2.7 and 1.9, respectively, where *f* values above 0.4 were considered to have a large effect size for one-way ANOVA.

**Fig. 3:**
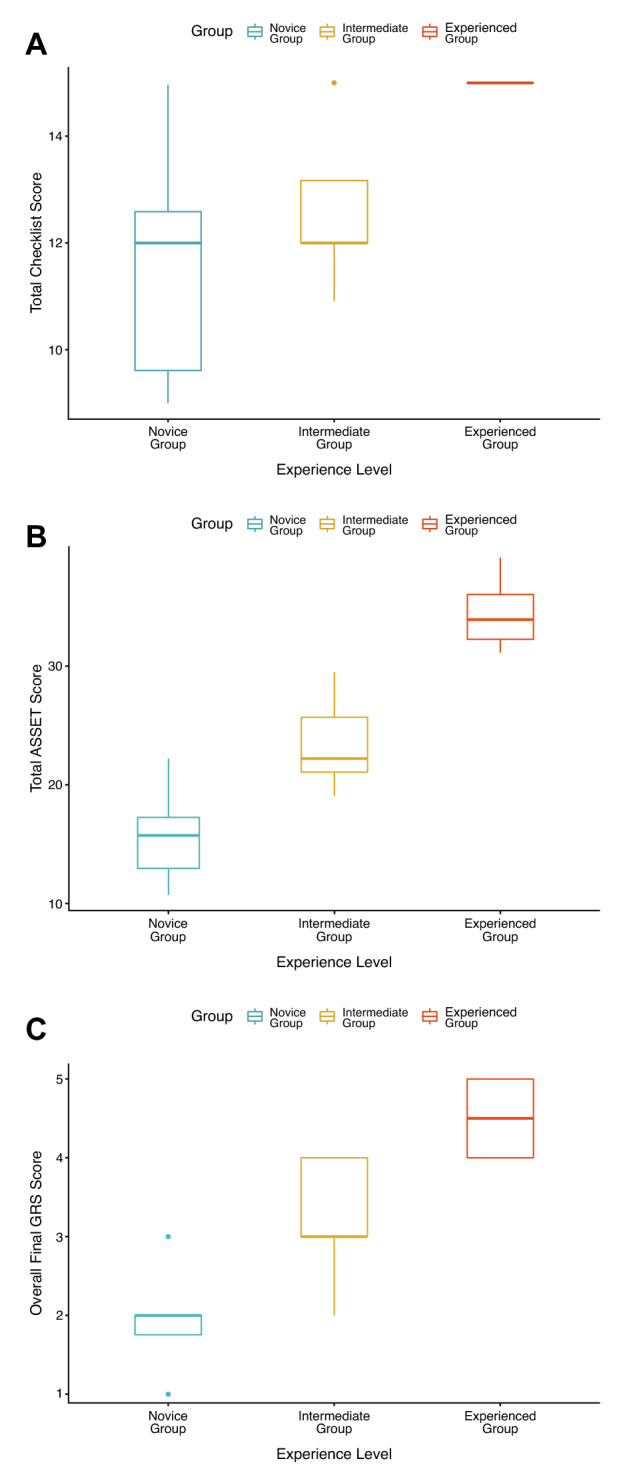
Box plots of the different assessment tools for the three groups with varying experience levels (Novice vs. Intermediate vs. Experienced). **A** Total checklist score; **B** Total Arthroscopic Surgical Skill Evaluation Tool (ASSET) score; **C** Final Global Rating Scale (GRS) score by experience level

**Fig. 4:**
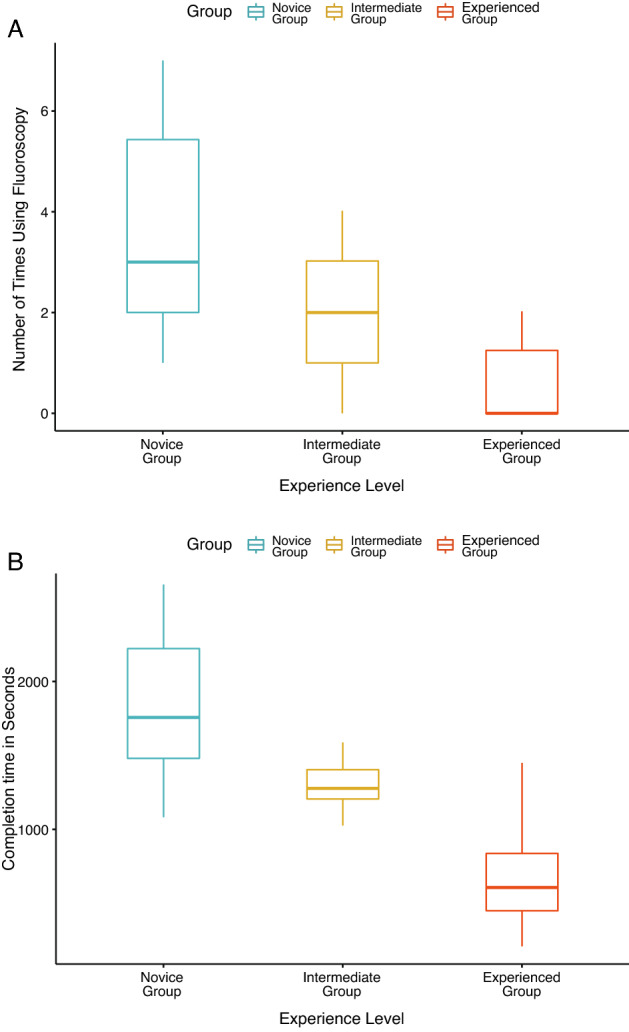
Box plots of task completion for the three groups with different experience levels (Novice vs. Intermediate vs. Experienced). **A** Number of times fluoroscopy was used; **B** Task completion time

One-way ANOVA revealed significant differences between the three subgroups with varying levels of experience in terms of the total checklist score (*F*_2,26_ = 11.3) (Fig. 3A), total ASSET score (*F*_2,26_ = 92.1) (Fig. 3B), overall final GRS score (*F*_2,26_ = 49) (Fig. 3C), number of times the participants used fluoroscopy (*F*_2,26_ = 7.4) (Fig. 4A) and task completion times (*F*_2,26_ = 23.5) (Fig. 4B). Positive correlations were observed between clinical experience and total ASSET score (hip arthroscopy *r* = 0.6; shoulder arthroscopy *r* = 0.5; knee arthroscopy *r* = 0.5). Negative correlations were seen between clinical experience and task completion time (hip arthroscopy *r* = − 0.6; shoulder arthroscopy *r* = − 0.5; knee arthroscopy *r* = − 0.5).

**Fig. 5:**
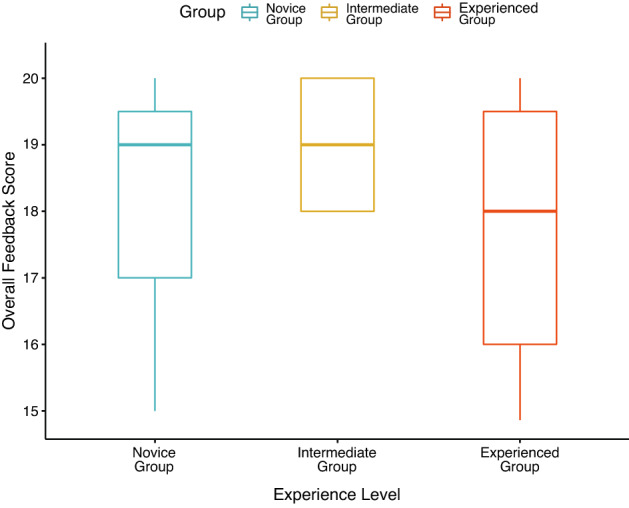
Box plot of the feedback scores collated from the three groups with varying experience levels (Novice vs. Intermediate vs. Experienced)

With regard to the feedback on the simulator, one-way ANOVA of the feedback scores given by the subgroups showed no significant differences in the feedback scores among the different groups (*F*_2,26_ = 1.5, *P* = n.s.) (Fig. 5). A post hoc Tukey test for pairwise comparison also revealed no significant differences in feedback scores between the groups. The mean score for each question in the feedback survey is shown in Table 2.

**Table 2:** Poststudy survey with mean scores for each question in the three groups

	Survey question	Novice group (mean, 95% CI)	intermediate group (mean, 95% CI)	Experienced group (mean, 95% CI)
1	Compared to real surgery, please grade the simulator’s anatomical accuracy under arthroscopy	4.3 [3.9, 4.8]	4.4 [3.9, 5]	4.2 [3.6, 4.8]
2	Compared to real surgery, please grade the haptic feedback of manipulating this simulator	4.2 [3.7, 4.6]	4.6 [4.1, 5]	4 [3.5, 4.5]
3	Do you think this simulator will help you to learn the skills for establishing arthroscopic portals of the hip?	5 [5]	4.8 [4.5, 5]	4.8 [4.4, 5]
4	Will you recommend using this simulator to practice establishing arthroscopic portals of the hip?	4.9 [4.8, 5]	5 [5]	4.8 [4.4, 5]

## Discussion

The most important finding of the present study was the development of a 3D printed simulator, which was proven to be a reliable assessment tool for evaluating orthopedic surgeons’ skills in performing hip arthroscopy. The combination of medical imaging, CAD and 3D-printing techniques has enabled the creation of a simulator, which can replicate hip arthroscopic techniques that include portal placement, intra-articular anatomical identification and arthroscope navigation. Based on the validation study, the participants’ performance in the simulated operation correlated with their experience level.

Many earlier studies have demonstrated the possibility of applying different types of simulations to benefit the hip arthroscopic field. The most common types of simulators include box trainers [1], virtual reality (VR)-based simulators [2, 4], and benchtop simulators [25, 26]. The box trainer is one of the simplest types of simulation and has hollow and geometric structures. It allows trainees to insert arthroscopic tools into its internal spaces to practice triangulation skills. It is also beneficial for beginners to gain familiarity with the use of arthroscopic tools. However, the downside is that because the box trainer does not contain any anatomical structures or provide any similar tactile feedback experienced during a real surgery, it cannot be used to simulate any surgical operations. The VR-based simulator is technologically advanced and can simulate varied skills with realistic visual and tactile feedback. However, certain important surgical skills, such as portal placement and screw fixation, cannot be replicated by the VR-based simulator. This means that trainees cannot hone the full suite of skills by using only the VR-based simulation. The benchtop simulator is very similar to the 3D printed simulator. Both are physical simulators that have accurate anatomical structures and are made with different materials to simulate varied layers of the human body. However, as benchtop simulators are mass produced, they do not have sufficient variations for simulating different clinical conditions. Unlike other types of simulators, the 3D printed simulator is created based on actual patient medical imaging data and a modular concept, providing great potential and flexibility to simulate different clinical conditions simply by replacing the modular components created based on different patient data. The ability to simulate specific disease conditions can help medical trainees become better prepared to meet the challenges associated with dynamic scenarios in the operating room.

The core function of the current 3D printed simulator, which is to simulate the operational skills related to portal placement, is unique and different from earlier studies that utilized preestablished portals [2, 4, 25, 26]. The 3D printed simulator can simulate the process of portal placement from skin positioning to insert the surgical tools with the guidance of fluoroscopy, making the process very similar to that in a real surgery scenario. Previously, cadaveric laboratories and operating rooms were considered the optimal environment for training or assessing these skills. However, the shortage of cadavers and operating room experience restricts the trainees’ learning efficiency and objectivity in competency assessment. This study has shown a useful alternative for hip arthroscopic surgeons to demonstrate their proficiency level outside the operating room.

Analysis of the data collated in the validation study shows that compared to participants with less experience in arthroscopy, more experienced participants tend to complete the simulated task in a shorter period of time, use fluoroscopy less frequently, and obtain higher scores in the task-specific checklist, ASSET and final GRS. These results affirm that the simulator can be used as an objective assessment tool to help orthopedic surgeons gauge the competence level of trainees’ surgical skills for performing hip arthroscopy.

According to the poststudy qualitative survey, the 3D printed simulator received favorable feedback from all the participants, and there were no significant differences in feedback scores among the three subgroups. The participants considered this simulator to be very helpful in the training of relevant skills needed for hip arthroscopic surgery. Most of the participants were satisfied with the fidelity of the simulator, with some expressing the opinion that the intra-articular anatomical structures and realistic tactile feeling of the soft tissue closely resemble that of the hip joint in the human body. A caveat is that participants in the novice and intermediate groups did not have experience performing hip arthroscopy. Hence, their ratings regarding the fidelity of the simulator were based on their prior learning and experience from other types of arthroscopic surgeries. In terms of anatomical accuracy and haptic feedback of the simulator, the response from the experienced group would be considerably more precise. The scores provided by the experienced group were relatively lower than those of the other two groups, indicating that from the perspective of experienced surgeons, the fidelity of the simulator can be further improved. This is not surprising since the structure of the simulator, such as the specific texture of the labrum, is not the same as that of the hip joint in the human body, and there are further considerations, such as the control of hemorrhage during actual surgery. Additionally, the soft tissue component in this simulator does not exhibit the different densities that simulate the various structural components of the hip joint. Learning in the operating room is still an indispensable part of a surgical training program.

As the validation study has proven that the 3D printed simulator can replicate critical steps in hip arthroscopy, there is a strong potential for this tool to be used routinely to assist the learning and practicing of relevant hip arthroscopic skills. This may help arthroscopic surgeons optimize their learning efficiency; moreover, the 3D printed simulator can be used for preliminary training, refresher courses and simulation of complex scenarios before actual surgeries, which would in turn mitigate the risk of medical errors in the operating room, hence enhancing patient safety. However, it remains to be seen whether arthroscopic surgery can in fact increase the proficiency of surgeons who are performing such techniques.

There are several limitations related to the 3D printed simulator and our validation study. First, the developed simulator can only simulate a limited number of surgical skills, and the operation can only be conducted in a dry environment, which differs from the setting in an operating room. Therefore, to practice the full suite of skills needed for hip arthroscopy with simulation-based training, arthroscopic surgeons will need to use different simulators. Second, for the sake of convenient usage, the outline of the simulator was not fully designed to mimic an anatomically accurate hip. Except for the curved surface of the operational field, the other surfaces were designed in the shape of a box. As a result, operating on the simulator is not fully intuitive and requires more guidance to be provided to the user. Third, as the sample size in this study is relatively small, it may be worthwhile to evaluate the utility of the simulator in a larger cohort of participants. Last, some of the assessment tools used in the validation study, such as the task-specific checklist and poststudy survey, were not validated previously and may have affected the accuracy of the results.

## Conclusion

The findings in this study demonstrate that based on the related techniques assessed, a reliable hip arthroscopic simulator can be developed for use by orthopedic surgeons to evaluate their hip arthroscopic skills before performing actual surgical operations.

## Supplementary Information

Below is the link to the electronic supplementary material.

Supplementary file1 (DOCX 18 KB)
