# Supplementary material for: A three-dimensional (3D) printed simulator as a feasible assessment tool for evaluating hip arthroscopy skills
Source: Knee Surg Sports Traumatol Arthrosc. 2022 Aug 29;31(5):2030–7. doi: 10.1007/s00167-022-07125-w (PMC10090017; doi:10.1007/s00167-022-07125-w)
Supplement: Supplementary file 1 — Supplementary file1 (DOCX 18 KB) [file 167_2022_7125_MOESM1_ESM.docx]

**Table 1 Task-Specific Checklist**

| **Tasks** | | **Yes** | **No** |
| --- | --- | --- | --- |
| **1** | Mark the surface projection of the anterior superior iliac spine and greater trochanter |  |  |
| **2** | Mark the operation area and the operation forbidden area |  |  |
| **3** | Mark the ALP, MAP, PMAP and DALA |  |  |
| **4** | Establish the ALP under fluoroscopy |  |  |
| **5** | Insert camera into ALP |  |  |
| **6** | Establish the MAP under the direct vision of arthroscopy (use c-arm fluoroscopy if necessary) |  |  |
| **7** | Put the probe into the articular cavity through the MAP |  |  |
| **8** | Observe the upper labrum through MAP, point out the labrum at 12 o'clock through ALP with probe |  |  |
| **9** | Observe the anterior labrum through ALP, point out the labrum at 2 o'clock through MAP with probe |  |  |
| **10** | Establish the DALA under the direct vision of ALP (use fluoroscopy if necessary) |  |  |
| **11** | Put the probe into the articular cavity through DALA |  |  |
| **12** | Observe the anterior inferior labrum. Point out the labrum at 4 o'clock through DALA with probe |  |  |
| **13** | Establish the PMAP under the direct vision of ALP (use fluoroscopy if necessary) |  |  |
| **14** | Put the probe into the articular cavity through the PMAP |  |  |
| **15** | Observe the upper labrum. Point out the labrum at 1 o'clock through the PMAP |  |  |
| Number of times fluoroscopy was used | |  | |

ALP indicates anterolateral portal, MAP indicates mid-anterior portal, PMAP indicates proximal mid-anterior portal, DALA indicates distal anterolateral portal.

**Table 2. Post Study Feedback Survey**

|  | **Survey Question** | | | | | |
| --- | --- | --- | --- | --- | --- | --- |
| **1** | Compared to real surgery, please grade the simulator’s anatomical accuracy under arthroscopy. | | | | | |
|  | 1  Very different | | 2 | 3 | 4 | 5  Very similar |
| **2** | Compared to real surgery, please grade the haptic feedback of manipulating this simulator. | | | | | |
|  | 1  Very different | | 2 | 3 | 4 | 5  Very similar |
| **3** | Do you think this simulator will help you to learn the skills of establishing arthroscopic portals of the hip? | | | | | |
|  | 1  Not at all | 2 | | 3 | 4 | 5  Very helpful |
| **4** | Will you recommend using this simulator to practice establishing arthroscopic portals of the hip? | | | | | |
|  | 1  Not at all | 2 | | 3 | 4 | 5  Absolutely |
